# Pomalidomide in patients with multiple myeloma: potential impact on the reconstitution of a functional T-cell immunity

**DOI:** 10.1007/s12026-024-09546-w

**Published:** 2024-09-24

**Authors:** Jiaxin Shen, Francesca Senes, Xiaofen Wen, Patrizia Monti, Shaoze Lin, Claudia Pinna, Andrea Murtas, Luigi Podda, Giuseppina Muntone, Gianni Tidore, Claudia Arru, Luca Sanna, Salvatore Contini, Patrizia Virdis, Leonardo Antonio Sechi, Claudio Fozza

**Affiliations:** 1 Department of Medicine, Surgery and Pharmacy, University of Sassari, Viale San Pietro 12, 07100 Sassari, Italy; 2 Department of Hematology, The First Affiliated Hospital of Shantou University Medical College, 515031 Shantou, P. R. China; 3 Department of Biomedical Sciences, University of Sassari, 07100 Sassari, Italy; 4 Department of Medical Oncology, Cancer Hospital of Shantou University Medical College, 515031 Shantou, P. R. China

**Keywords:** Pomalidomide, Relapsed/refractory multiple myeloma, Flow cytometry, CDR3 spectratyping, T-cell repertoire, Antitumor immunity

## Abstract

**Background:**

Pomalidomide, a third-generation oral immunomodulatory drug, exhibits efficacy in patients with relapsed multiple myeloma or those refractory to bortezomib and lenalidomide (RRMM).

**Methods:**

In this clinical context, we employed flow cytometry and CDR3 spectratyping to monitor the dynamics of the T-cell repertoire during Pomalidomide treatment, aiming to investigate its potential to reverse the immunological abnormalities characteristic of RRMM.

**Results:**

By flow cytometry at baseline we found a significant decrease in CD4 + frequency in MM patients, while CD8 + frequency were significantly higher in patients when compared to controls. Most T cell populations remained stable across all time points, except for CD4 + frequency, which notably decreased from t1 to subsequent assessments. Our investigation revealed as most relevant finding the notable increase in CD4 + expansions and the growing prevalence of patients manifesting these expansions. This pattern is even more evident in patients receiving their treatment until t3 and therefore still responding to treatment with Pomalidomide. We also conducted a comparison of spectratyping data before and after treatment, substantially demonstrating a relatively stable pattern throughout the course of Pomalidomide treatment.

**Conclusions:**

These observations imply that Pomalidomide treatment influences the T-cell repertoire, particularly in the CD4 + subpopulation during the later stages of treatment, raising speculation about the potential involvement of these lymphocyte expansions in mechanisms related to antitumor immunity.

**Supplementary Information:**

The online version contains supplementary material available at 10.1007/s12026-024-09546-w.

## Introduction

Multiple myeloma (MM) is a hematological malignancy marked by the clonal expansion of plasma cells, originating from mature post-follicular B cells, that produce monoclonal immunoglobulin (M-protein) [1]. MM is the second most common blood cancer in high-income countries, with an annual incidence of 4.5 to 6 cases per 100,000 individuals, primarily diagnosed in individuals around 70 years old [2]. Patients often present with an asymptomatic precursor condition, monoclonal gammopathy of undetermined significance (MGUS). In the absence of the hallmark CRAB events—hypercalcemia, renal insufficiency, anemia, and bone lesions—associated with MM, patients can progress to smoldering multiple myeloma (SMM) [3]. When organ dysfunction appears, the condition may progress to active myeloma. The progression of MM is strongly influenced by the bone marrow microenvironment (BMME), which supports myeloma cell survival through signals from infiltrating inflammatory cells [4]. Understanding this interaction has led to significant therapeutic advances.

In recent years, treatments for MM have substantially improved, particularly with the introduction of immunomodulatory drugs (IMiDs) like thalidomide and lenalidomide, proteasome inhibitors, and monoclonal antibodies. Despite these advances, the prognosis for relapsed or refractory MM (RRMM) remains poor [5]. Early studies suggest that IMiDs may restore immune synapse function, especially in chronic lymphocytic leukemia (CLL) [6]. Early studies suggest that IMiDs may restore immune synapse function, especially in chronic lymphocytic leukemia (CLL) [7]. Research shows that Pomalidomide enhances T-cell function and numbers by engaging the Cereblon/Ikaros pathway, which is critical for immune activation [8].

Immunomodulatory therapies are key to cancer treatment, with T lymphocytes playing a central role in coordinating anti-tumor immune responses by recognizing tumor antigens [9]. The diversity of the T-cell receptor (TCR) repertoire reflects the range of T-cell responses within the immune system [10]. Monitoring the TCR repertoire non-invasively in peripheral blood provides valuable insights into immune function in cancer patients [11]. This allows researchers to analyze T-cell clones in various contexts, offering insights into immune responses during different stages of disease [12–14]. In MM, patients often exhibit a skewed TCR repertoire and impaired T-cell function, characterized by a reduced CD4+/CD8 + ratio due to diminished CD4 + T-cell levels and an increase in CD8 + T cells [15–17].

Evaluating changes in T-cell clone distribution and TCR diversity during treatment can offer crucial insights into therapeutic efficacy, disease progression, and recurrence risk [18]. The complementarity-determining region 3 (CDR3), which undergoes random rearrangements and mutations in the V(D)J regions, is critical in T-cell recognition of antigens [19]. Studies in solid tumors have demonstrated that TCR and CDR3 diversity are important for diagnosis, treatment, and prognosis [20–24]. In MM, CDR3 sequences can serve as stable, patient-specific markers in minimal residual disease (MRD) studies [25, 26]. CDR3 spectratyping, a polymerase chain reaction (PCR)-based technique, is highly sensitive in detecting clonal T-cell expansion and provides detailed insights into T-cell diversity across immune-mediated and malignant conditions [27, 28].

Despite advancements in MM treatment, longitudinal studies examining the TCRα and TCRβ repertoire changes during therapy remain limited. Such studies are crucial for understanding the role of T cells in MM progression and therapeutic response. In this study, we hypothesize that Pomalidomide treatment in RRMM patients will not only influence disease progression but also restore functional T-cell immunity. Specifically, we predict that Pomalidomide will increase the diversity and clonal distribution of the TCR repertoire, particularly within CDR3, and improve the balance and function of T-cell subsets. These changes may reflect improved immune surveillance and tumor control. Our study aims to monitor these effects using a non-invasive approach, providing insights into the immunological mechanisms of Pomalidomide and its potential for enhancing patient outcomes.

## Materials and methods

### Patients

The analysis encompassed 40 participants, including 10 patients with relapsed/refractory multiple myeloma (RRMM) and 30 healthy controls, enrolled between February 16, 2016, and November 19, 2018. Patient characteristics are provided in Table 1. Eligibility criteria for inclusion were: (1) Age ≥ 18 years, (2) Histologically or pathologically confirmed diagnosis of multiple myeloma (MM), and (3) First diagnosis of MM. Exclusion criteria included: (1) Incomplete clinical or pathological data, (2) Recent blood transfusions, (3) Use of hematopoietic-promoting drugs, or (4) Ongoing or recent severe infections. Patients underwent an initial evaluation at baseline (T1), followed by assessments every three cycles of Pomalidomide treatment. The study was approved by the local ethics committee (approval number 2436/2016). The diagnosis of MM was confirmed through bone marrow aspiration, biopsy, and peripheral blood counts, with staging based on the International Staging System (ISS). Additional details regarding ISS classification, time intervals from initial therapy to Pomalidomide, with other characteristics of the patients and information about sample collection are available in Supplementary Table 1.

**Table 1:** Patients’ group composition

Characteristics	
N	10
Age, Median (years)	70.5
Sex	
Male	5
Female	5
Diagnosis Multiple Myeloma (ISS)	
I	2
II	5
III	3
Cytogenetic	
Normal	8
NA	2
Treatment regimen	
Pomalidomide	10

### Flow-cytometry

Initially, we determined the frequency of various cell subsets in peripheral blood using antibodies targeting CD3, CD4, CD8, CD16, and CD56. Following this, we examined the frequency of regulatory T-cells by identifying the CD4 + cell subset characterized by exceptionally high surface expression (> 2 log) of CD25 and very low expression of CD127 (< 2 log). All antibodies used were sourced from Becton Dickinson (San Jose, CA [29]), as detailed in Supplementary Table 2.

To assess the T-cell receptor-beta variable (TCR-BV) repertoire, we employed the IOTest Beta Mark Kit (Beckman Coulter, San Diego, CA) for flow-cytometric analysis, following the manufacturer’s instructions. The samples were acquired using a FACS Canto flow cytometer with FACS Diva software, and we established a lymphocyte gate based on forward and side scatter characteristics. The relative representation of a specific BV family was expressed as the percentage of cells stained with the family-specific antibody within the CD4 + or CD8 + cell population. BV expansion was defined as any value of BV family expression exceeding the mean + 3 standard deviations (SD) calculated from normal control samples. It’s worth noting that the BV values obtained from normal controls were previously compared with those reported in the literature [30], demonstrating similar expression levels and justifying their use in defining the normal range.

### CD4 + and CD8 + cell separation

Peripheral blood monocytes were isolated using gradient centrifugation with Ficoll-Hypaque (Sigma Diagnostic, St. Louis, USA). The cells were then separated into CD4 + and CD8 + subsets through positive selection, utilizing antibody-coated immunomagnetic beads (Dynabeads; Dynal, Oslo, Norway) for 30 min at 4 °C on a rotating shaker, followed by magnetic isolation. TRIzol (Invitrogen, Paisley, UK) was subsequently added to the cell pellets, which were then stored at -80 °C until RNA extraction. Details on the antibodies and their clones are provided in Supplementary Tables 2, and the gating strategy is illustrated in Supplementary Fig. 1.

### CDR3 spectratyping

RNA extraction was carried out from each cell subset, and complementary DNA synthesis followed the manufacturer’s instructions (Invitrogen, Paisley, UK). Subsequently, PCR was conducted to amplify the 24 TCR-BV regions expressed in the samples. The sequences of the TCR-BV and BC primers were described in the literature [31]. Following PCR amplification, the BV fragments were subjected to analysis on an ABI Prism 3500 Genetic Analyzer (Applied Biosystems, Foster City, CA). Data acquisition and analysis were performed using the ABI Prism GeneMapper Analysis software version 3.0 (Applied Biosystems, Foster City, CA).

### Spectratyping analysis

To analyze spectratyping data, we dissected the profiles based on peak area and shape to determine the degree of skewing (S) and oligoclonality (O) [32]. A profile was considered normal (G) if it exhibited a Gaussian bell-shaped distribution, featuring distinct peaks spaced by three nucleotides. To detect evidence of oligoclonal expansion or skewing within each BV, we calculated the relative fluorescence intensity (RI) for each peak (RI = peak area/total BV peak area).

A profile was classified as skewed if it met any of the following criteria: (a) it contained a dominant peak with an RI greater than 50% of the total peak area; (b) two dominant peaks were present, and the RI of each peak exceeded 25% of the total peak area; or (c) multiple peaks deviated from a Gaussian pattern, and the RI of dominant peaks was higher than 25% of the total peak area. Criterion (a) was specifically used to identify oligoclonal BVs. The percentages of skewed and oligoclonal BV subfamilies were then calculated among the total number of BVs analyzed for each patient.

### Statistical analysis

The patient sample sizes varied across time points, with 10 patients at T1, 9 patients at T2, 7 patients at T3, and 5 patients at T4. To facilitate comparison and account for the varying patient numbers, the data were primarily presented as percentages rather than absolute frequencies. A control group consisting of 30 individuals matched for age and sex was compared to the pre-treatment patient group, and the statistical analysis was performed using the Mann-Whitney Test. For comparisons between different time points, the Friedman test was initially applied, followed by the Wilcoxon signed-rank test. The threshold for statistical significance was set at *p* < 0.05.

## Results

### T-cell frequencies determined by flow cytometry before and during pomalidomide treatment

**Fig. 1:**
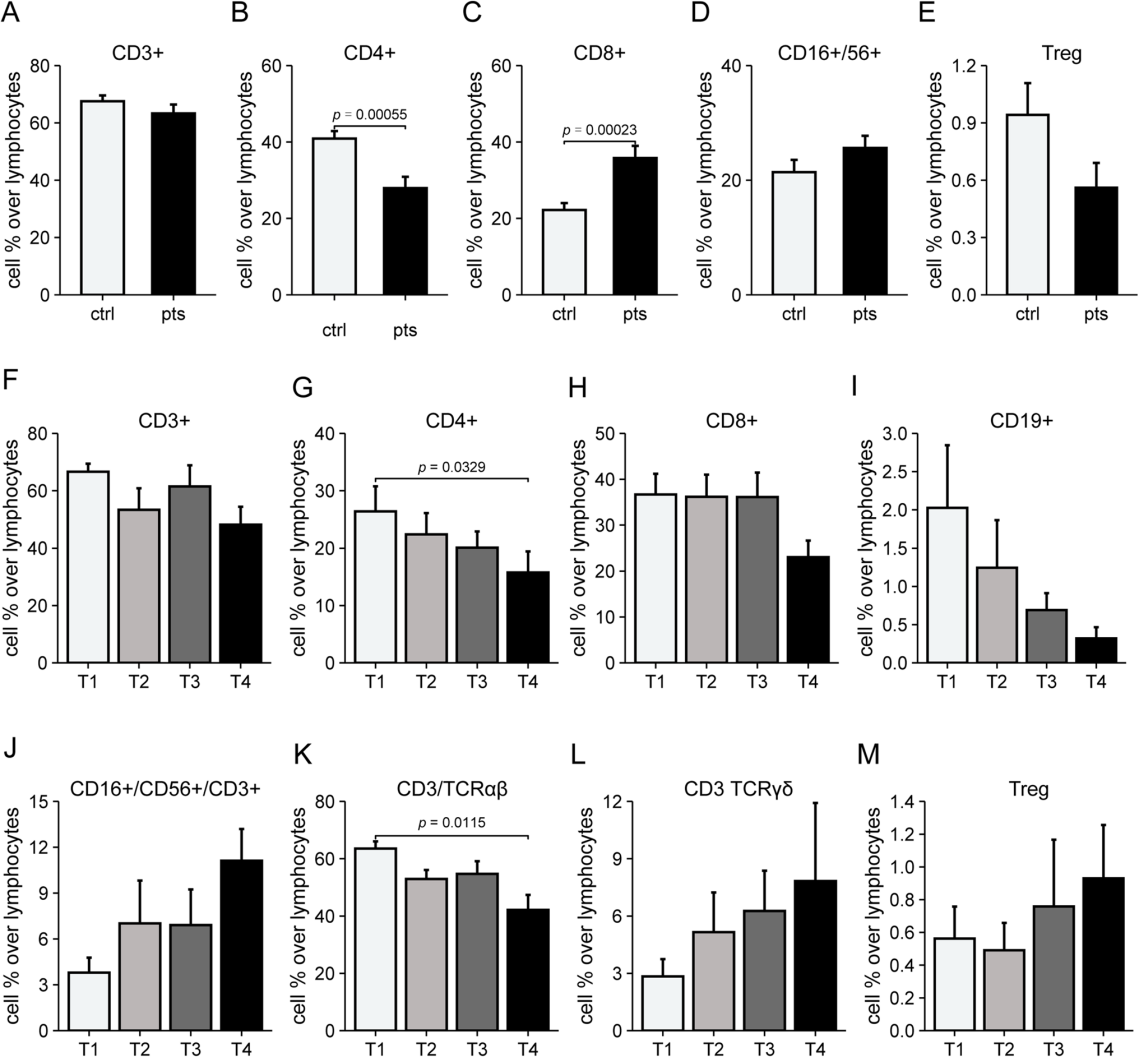
T-cell frequencies determined by flow cytometry before and during pomalidomide treatment. (**A**-**E**) T-cell frequencies of a pool of age- and sex- matched healthy control (ctr) that were compared with MM patients (pts) prior to initiating Pomalidomide treatment. The lymphocytes subpopulations are reported as percentage over total lymphocyte population from peripheral blood samples collected at t = 1. Statistical difference between the two groups (Ctl vs. Pts) is evaluated with the Mann Whitney test (two-tailed, non-parametric test for non-matched samples). (**F**-**M**) The percentages of lymphocyte subpopulations in MM patients are shown relative to the total lymphocyte population in peripheral blood samples collected before Pomalidomide treatment and at 3, 6, and 12 months (t1, t2, t3, and t4, respectively). Statistical difference within these groups is evaluated with the Friedman test (non-parametric test for repeated measures)

Our patient group was preliminary compared with a group of sex and age matched healthy people before starting Pomalidomide. We first assessed the frequencies of CD3+, CD4+, CD8+, and CD16-56 + cells, as illustrated in Fig. 1A-E. Intriguingly, we found a significant decrease in CD4 + frequency (mean 40.9% vs. 27.9%, *p* = 0.00055) in MM patients, while CD8 + frequency (mean 22.2% vs. 35.8%, *p* = 0.00023) were significantly higher in patients when compared to controls. The frequency of CD3+ (mean 67.6% vs. 63.3%, *p* = 0.27), CD16+/56+ (mean 21.4% vs. 25.62%, *p* = 0.21) and Treg (mean 0.94% vs. 0.56%, *p* = 0.12) cells was substantially stable, without showing any significant difference.

Our patients underwent a median of three assessments during their treatment with Pomalidomide, corresponding to t2, t3 and t4. We tracked the frequencies of CD3+, CD4+, CD8+, CD19+, CD3+/16+/56+, CD3+/TCRαβ, CD3+/TCRδλ, and Treg, during therapy, as depicted in Fig. 1F-M. Most cell populations remained stable across all time points, except for CD4 + frequency, which notably decreased from t1 to subsequent assessments (t2, t3, and t4; *p* = 0.0329). A similar declining trend was observed in CD3+/TCRαβ frequency (*p* = 0.0115), suggesting that TCR rearrangements indeed occurred during the course of Pomalidomide treatment. However, this trend was not evident in B-lymphocytes (CD19 + cells; *p* = 0.0538). The frequencies of natural killer T cells (CD3+/16+/56+, *p* = 0.9444) and Treg (*p* = 0.9282) exhibited an upward trend, despite the absence of statistical significance.

### Kinetic of expanded T-cell subpopulations in MM patients during pomalidomide treatment

**Fig. 2:**
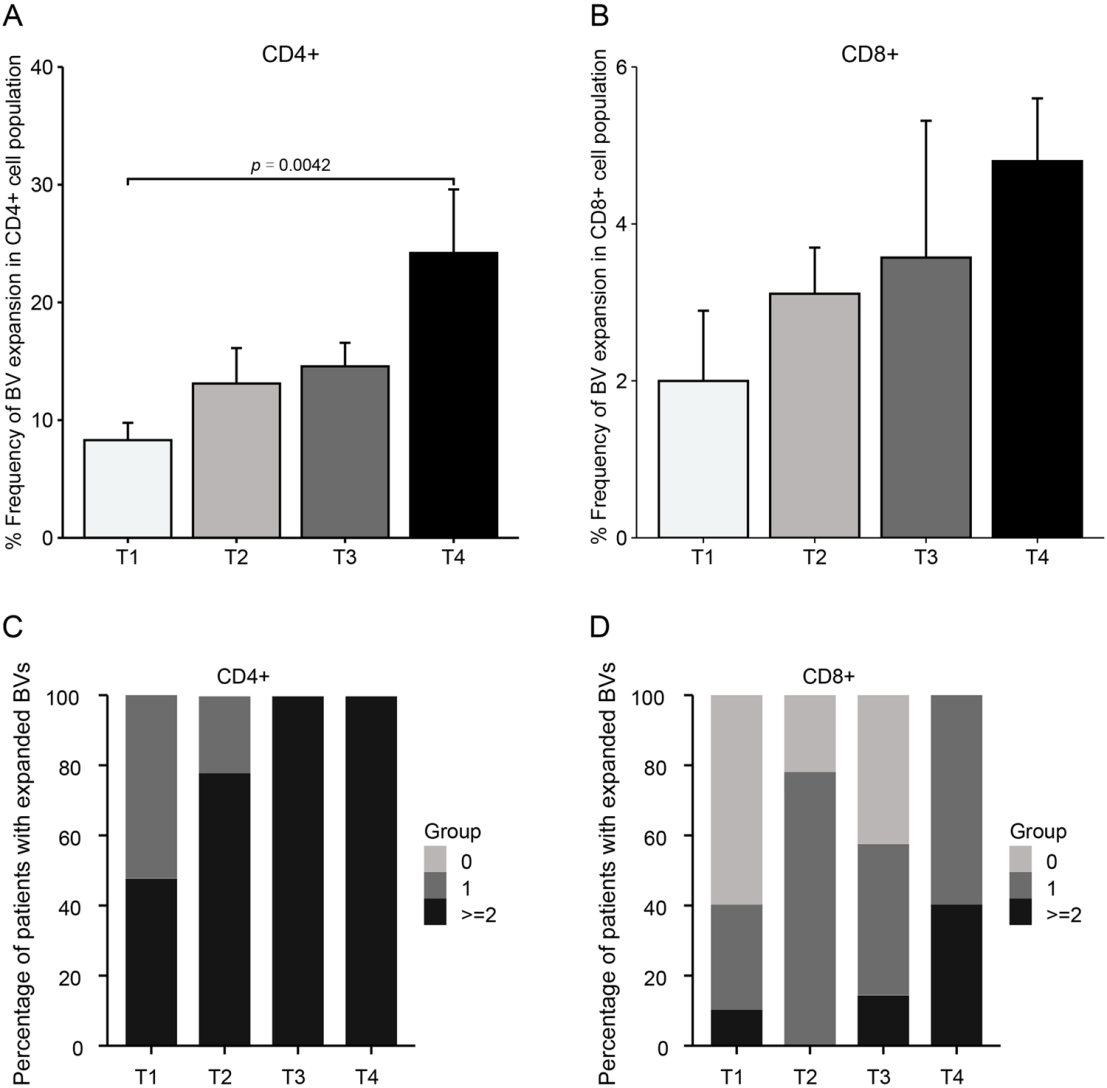
Kinetic of expanded T-cell subpopulations in MM patients during Pomalidomide treatment. (**A**, **B**) Percentages of BV expansions over total BV examinate from CD4 + subpopulation and from CD8 + subpopulation of MM patients. Peripheral blood samples were collected before Pomalidomide treatment and after 3-, 6- and 12-months therapy including Pomalidomide (t1, t2, t3 and t4 on x-axis, respectively) (**C**, **D**) Percentages of MM patient that show none, a single or at least two expansions of BVs from CD4 + subpopulation and from CD8 + subpopulation. Peripheral blood samples were collected before Pomalidomide treatment and after 3-, 6- and 12-months therapy including Pomalidomide (t1, t2, t3 and t4 on the x-axe, respectively)

We further examined the expansion of T-cell subpopulations in MM group during Pomalidomide treatment. We noted that the BV expansion percentages in the CD4 + subpopulation relative to the total BV examined were 8%, 13%, 14%, and 24% for t1, t2, t3, and t4, respectively **(**Fig. 2A**)**. In more detail, seven out of ten patients showed an overall increasing trend of BV expansions, with their proportion ranging from 4 to 38%. This trend was also consistent with their response to treatment, as we collected in Supplementary Table 1. However, three expansions disappeared in subsequent evaluations, while three patients displayed stable or slightly decreased BV expansion during treatment. While in the CD8 + subpopulation, the percentages of BV expansions relative to the total BV examined were 2%, 3%, 4%, and 5%, also corresponding to t1, t2, t3, and t4, respectively **(**Fig. 2B**)**. Six out of ten patients with baseline BV expansions displayed an increased proportion, ranging from 2 to 8%. Unlike CD4+, BV expansions in CD8 + remained relatively stable during treatment, with three patients maintaining similar levels compared to previous assessments. This suggests that BV expansion in CD8 + cells during treatment is typically transient. In general, there was a consistent upward trend in BV expansions in both CD4 + and CD8 + subpopulations, aligning with the expected induction of CD4 + and CD8 + cells by Pomalidomide. The very limited numbers of patients would be one of the key reasons that we could not show any relevant difference in both CD4 + and CD8 + cells during treatment.

Next, we observed the distribution of patients with none (0), a single (1), or 2 or more BV expansions over time in CD4 + and CD8 + cells **(**Fig. 2C, D**)**. A key observation emerging from our study was the notable increase in CD4 + expansions and the rising incidence of patients demonstrating these expansions. Specifically, in relation to CD4 + cells, the distribution was as follows: single or multiple BV expansions accounting for all patients at t1. At t2, the distribution shifted to single expansions for the minority (22%), while the majority exhibited multiple expansions (78%). Remarkably, at t3 and t4, all patients displayed multiple expansions (100%). Similarly, for CD8 + cells, the proportions were as follows: multiple expansions for 60% of patients, single expansions for 30% of patients, and multiple expansions for the rest at t1. By t2, the scenario changed, with a higher occurrence of single expansions in almost 80% of patients. Following this trend, BV expansions were observed to experience a more dramatic increase, from nearly 60% (t3) to 100% of patients (t4).

Overall, our analysis revealed a consistent trend that an increasing fraction of patients harboring at least one expansion by the end of the monitoring period. Notably, for CD4 + cells, all patients manifested at least two expansions starting from t3, prompting speculation about the potential involvement of these lymphocyte expansions in mechanisms associated with antitumor immunity.

### Percentages of CDR3 skewing (skewed) and oligoclonality (oligo) in both CD4 + and CD8 + cells

**Fig. 3:**
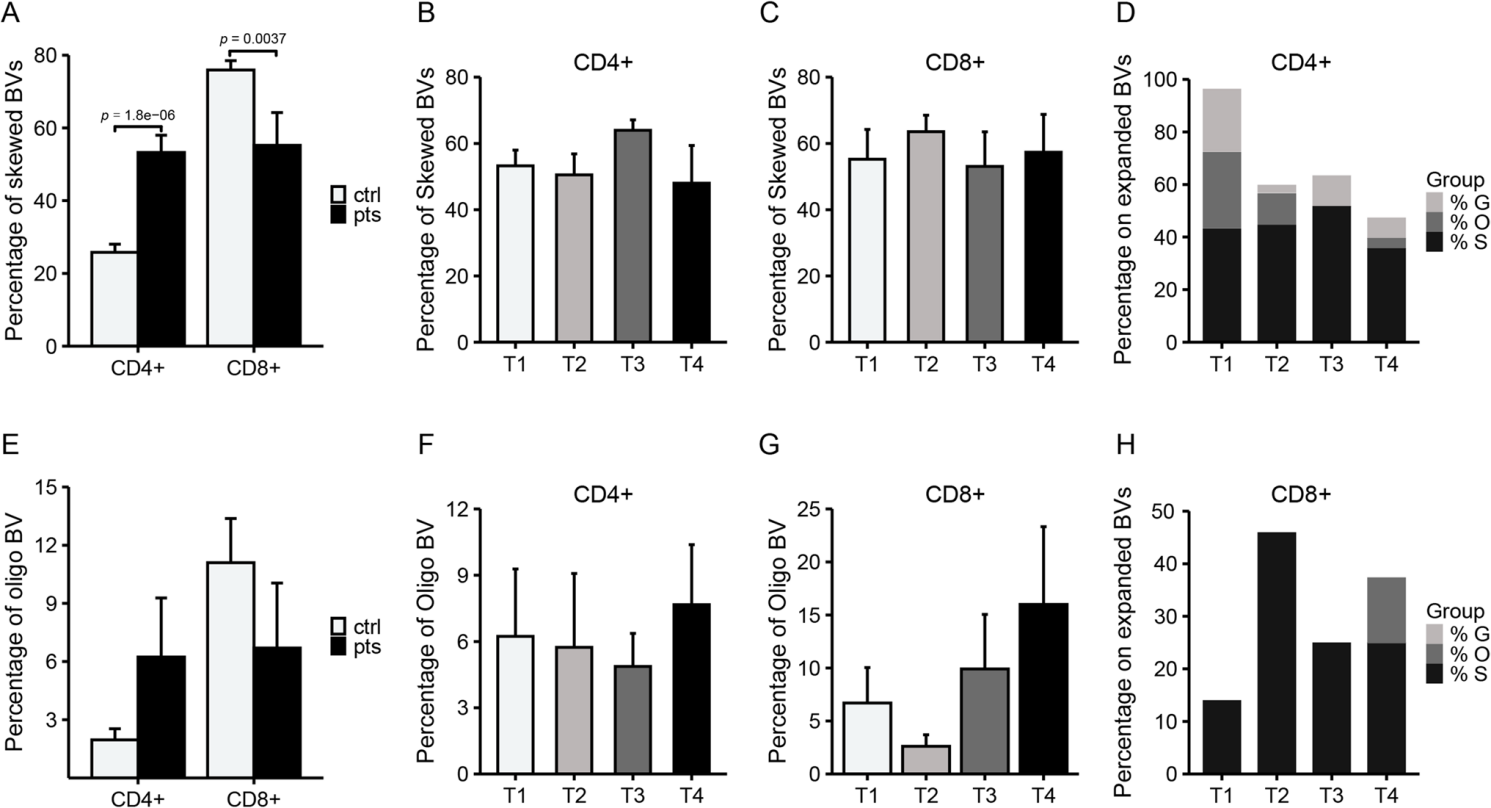
Percentages of CDR3 skewing (Skewed) and oligoclonality (oligo) in both CD4 + and CD8 + cells. (**A**, **E**) Percentages of skewed and oligo BVs were determined by spectratyping of a pool of age- and sex- matched healthy controls (ctr) with MM patients (pts) before starting Pomalidomide therapy. Statistical difference between groups is calculated with Mann Whitney test (non-parametric test for unpaired measures) (**B**, **C**, **F**, **G**) Percentages of skewed and oligo BVs were determined by spectratyping of samples before Pomalidomide treatment (t1) and after 3-, 6- and 12-months therapy including Pomalidomide (t2, t3 and t4 on x-axis, respectively). Statistical difference within groups is calculated with Friedman test (non-parametric test for repeated measures) (**D**, **H**) Percentages of CDR3 skewing (**S**), oligoclonality (**O**) and Gaussian profile (**G**) were found among BV expanded from both CD4 + and CD8 + cells. Data are reported for samples were collected before Pomalidomide treatment (t1) and after 3, 6 and 12 months of therapy including Pomalidomide (t2, t3 and t4 on x-axis, respectively). Percentages were obtained by formulae: % G on exp: (Number of Gaussian/number of total expanded BVs) * 100; % O on exp: (Number of oligo/number of total expanded BVs) *100; % S on exp: (Number of Skewed/number of total expanded BVs) * 100

During Pomalidomide treatment, we employed spectratyping to monitor changes in CDR3 profiles in our patients, with more evident changes observed in the CD4 + subpopulation. Initially, we conducted a comparative analysis between patients and controls **(**Fig. 3A**)**. In the CD4 + population, we observed a higher degree of CDR3 skewing in CD4 + cells among patients compared to controls (mean 25.8% vs. 55.3%, *p* < 0.0001). Conversely, the percentage of skewed BVs was significantly decreased in patients in CD8 + population (mean 75.9% vs. 55.2%, *p* < 0.0001), indicating a reduction in skewing profiles in the CD8 + population among patients compared to controls. However, as indicated in Fig. 3E, the percentage of oligoclonal BV subfamilies was consistent between patients and controls, both in the CD4+ (mean 1.97% vs. 6.22%, *p* = 0.1798) and in the CD8 + subset (mean 11.1% vs. 6.67%, *p* = 0.5340).

We continued to observe the kinetics of CDR3 skewing and CDR3 oligoclonality in CD4 + and CD8 + cells at various assessment points before and during their treatment with Pomalidomide (t1, t2, t3, and t4). In CD4 + cells, CDR3 skewing values were 53.2%, 50.5%, 64.0%, and 48.0%, respectively **(**Fig. 3B**)**. Meanwhile, the CDR3 skewing values in CD8 + cells were 55.2%, 63.5%, 53.1%, and 57.4%, respectively **(**Fig. 3C**)**. The kinetics of CDR3 oligoclonality in CD4 + cells were 6.23%, 5.73%, 4.87%, and 7.67%, respectively, while in CD8 + cells, they were 6.70%, 2.60%, 9.9%, and 16.0%, respectively **(**Fig. 3F, G**)**. We also conducted a comparison of these spectratyping data before and after treatment, once again demonstrating a relatively stable pattern throughout the course of Pomalidomide treatment.

Finally, the study specifically examined clonal expansions within CD4 + and CD8 + cells, categorized into Skewed, Oligoclonal, and Gaussian. Across the treatment phases, notable shifts in proportions were observed. In CD4 + cells, the prevalence of Skewed increased from 43% (t1) to 52% (t3), then decreased to 36% (t4). Concurrently, the proportion of Gaussian decreased from 24% (t1) to 12% (t3) and further to 8% (t4). Oligoclonal expansions exhibited variability, declining from 29% (t1) to 0% (t3), followed by a marginal resurgence to 4% (t4) (Fig. 3D**)**. Regarding CD8 + cells, the prevalence of Skewed ascended from 14% (t1) to 46% (t2), subsequently stabilizing at 25% at both t3 and t4. Oligoclonal expansions remained absent except for a resurgence to 12.5% at t4 **(**Fig. 3H**)**. It is crucial to acknowledge the presence of data gaps and expansions that do not neatly fit into the designated categories (S, O, or G), thus resulting in percentages that do not sum up to 100%.

## Discussion

The introduction of novel pharmaceutical agents has significantly expanded the treatment options for Multiple Myeloma (MM) patients. Despite these advancements and extended periods of remission, MM remains incurable, with relapse being a frequent outcome. Each relapse typically worsens patient prognosis, leading to increased frailty, complications, reduced tolerance to treatment-related toxicities, higher cytogenetic abnormalities, and diminished immune functionality [5, 33]. Consequently, selecting treatments for Relapsed/Refractory Multiple Myeloma (RRMM) involves careful consideration of patient-specific factors, disease characteristics, and treatment regimens.

Pomalidomide has emerged as a pivotal therapeutic agent due to its unique mechanism involving the Cereblon protein (CRBN). As a third-in-class immunomodulatory drug (IMiD), Pomalidomide operates through three main mechanisms: direct antitumor effects (including antiproliferative and pro-apoptotic effects), modulation of the bone marrow microenvironment (such as antiangiogenic and anti-inflammatory effects), and immunomodulation (including activation of NK and T-cells and T-cell co-stimulation) [34, 35]. Its significance in treating RRMM is underscored by numerous international guidelines, including those from the Mayo Clinic [36], the European Hematology Association (EHA) and the European Society for Medical Oncology (ESMO) [37], the International Myeloma Working Group [38], and National Comprehensive Cancer Network (NCCN) [39]. These guidelines consistently endorse Pomalidomide as a cornerstone of RRMM therapy.

Our study provides additional insights into Pomalidomide’s potential for immune modulation in MM patients. We found that treatment with Pomalidomide, often combined with high-dose steroids, led to a significant increase in CD4 + T-cell expansions and a higher prevalence of patients exhibiting these expansions. This pattern was particularly pronounced in patients continuing treatment at the third time point, suggesting these expansions may contribute to antitumor immunity (Supplementary Table 1). One possible explanation is the observed Pomalidomide-induced degradation of transcriptional suppressors, such as Kiaros and Aiolos, which interact with CRBN and are linked to T-cell activation [40]. The increase in CD4 + T-cells highlights their important role in mediating antitumor immunity, in contrast to the direct tumor-killing effects of CD8 + T-cells [41]. Furthermore, Pomalidomide enhances T-cell co-stimulation, boosting the production of Th1 cytokines and activating NK and NKT cells [42, 43]. Dynamic changes in Tregs during Pomalidomide treatment were also noted. The exact role of Tregs in MM is still under investigation; studies by Mutu et al. have shown that functionally active Tregs correlate with poor clinical outcomes and increased progression risk [44]. However, conflicting results exist, with some research indicating that IMiDs, including Pomalidomide, increase Tregs, especially those expressing CD38 [45]. These discrepancies may arise from factors such as the primary site (PB or tumor), concurrent dexamethasone dosage, patient selection, and differing definitions of Tregs [46]. This variability highlights the need for a direct comparative analysis of pharmacodynamic properties to optimize Pomalidomide’s use in MM treatment.

Further evidence supports Pomalidomide’s efficacy in reversing immune dysfunction associated with MM, such as impaired T-cell distribution and reduced peripheral blood CD4 + and CD8 + T-cells, partly due to TGF-β secretion [16, 47]. These discoveries shed light on new perspectives for pharmaceutical IMiDs within BMME. Noteworthy in vitro studies have underscored the robust immune modulation effects of IMiDs. For instance, Pomalidomide has exhibited an astonishing 300-1200-fold increase in efficacy at enhancing T cell proliferation, leading to increased secretion of interleukin-2 (IL-2) and interferon-gamma (IFN-γ) [48]. These findings provide a comprehensive understanding of IMiDs’ clinical benefits and suggest valuable insights for optimizing treatment strategies and developing rational combination therapies.

We acknowledge that the smaller sample size in this study may have limited our statistical power, potentially affecting our ability to detect certain differences. We appreciate the participation of eligible individuals and recognize that smaller sample sizes are common in exploratory studies. To address this limitation, we plan to expand the patient cohort and refine our research methodology in future studies. Initially, we employed techniques such as spectratyping [49, 50] and flow cytometry [51, 52] to detect oligoclonal T-cell expansions and monitor clonal sizes of CDRβ3s. However, these methods did not provide insights into TCR similarity [53]. With the advent of high-throughput sequencing (HTS), we can now achieve more precise characterization of adaptive immune receptors, offering information on both clone sizes and sequence relatedness. This advancement underscores the need for effective summary measures to interpret the extensive data generated by these techniques.

## Supplementary Information

Below is the link to the electronic supplementary material.

ESM 1 (DOCX 221 KB)

ESM 2 (DOCX 22.0 KB)

ESM 3 (DOCX 18.4 KB)

## Data Availability

All data supporting the findings of this study are available within the paper and from the corresponding author upon reasonable request.

## References

[1] Kumar SK, et al. Multiple myeloma. Nat Rev Dis Primers. 2017;3:17046. 28726797 10.1038/nrdp.2017.46

[2] van de Donk N, Pawlyn C, Yong KL. Multiple myeloma. Lancet. 2021;397(10272):410–27. 33516340 10.1016/S0140-6736(21)00135-5

[3] Ho M, et al. Changing paradigms in diagnosis and treatment of monoclonal gammopathy of undetermined significance (MGUS) and smoldering multiple myeloma (SMM). Leukemia. 2020;34(12):3111–25. 33046818 10.1038/s41375-020-01051-x

[4] Neumeister P, et al. Targeting the microenvironment for treating multiple myeloma. Int J Mol Sci. 2022;23(14):7627. 35886976 10.3390/ijms23147627 PMC9317002

[5] Kastritis E, Terpos E, Dimopoulos MA. How I treat relapsed multiple myeloma. Blood. 2022;139(19):2904–17. 35007326 10.1182/blood.2020008734

[6] Ramsay AG, et al. Chronic lymphocytic leukemia T-cells show impaired immunological synapse formation that can be reversed with an immunomodulating drug. J Clin Invest. 2008;118(7):2427–37. 18551193 10.1172/JCI35017 PMC2423865

[7] Richardson PG, et al. Pomalidomide, bortezomib, and dexamethasone for patients with relapsed or refractory multiple myeloma previously treated with lenalidomide (OPTIMISMM): a randomised, open-label, phase 3 trial. Lancet Oncol. 2019;20(6):781–94. 31097405 10.1016/S1470-2045(19)30152-4

[8] Zhu YX, et al. Identification of cereblon-binding proteins and relationship with response and survival after IMiDs in multiple myeloma. Blood. 2014;124(4):536–45. 24914135 10.1182/blood-2014-02-557819 PMC4110660

[9] Gonzalez H, Hagerling C, Werb Z. Roles of the immune system in cancer: from tumor initiation to metastatic progression. Genes Dev. 2018;32(19–20):1267–84. 30275043 10.1101/gad.314617.118 PMC6169832

[10] Nikolich-Zugich J, Slifka MK, Messaoudi I. The many important facets of T-cell repertoire diversity. Nat Rev Immunol. 2004;4(2):123–32. 15040585 10.1038/nri1292

[11] Sims JS, et al. Diversity and divergence of the glioma-infiltrating T-cell receptor repertoire. Proc Natl Acad Sci USA. 2016;113(25):E3529–37. 27261081 10.1073/pnas.1601012113 PMC4922177

[12] Strønen E, et al. Targeting of cancer neoantigens with donor-derived T cell receptor repertoires. Science. 2016;352(6291):1337–41. 27198675 10.1126/science.aaf2288

[13] Pan RY, et al. Identification of drug-specific public TCR driving severe cutaneous adverse reactions. Nat Commun. 2019;10(1):3569. 31395875 10.1038/s41467-019-11396-2 PMC6687717

[14] Liang Q, et al. Intrahepatic T-Cell receptor β immune repertoire is essential for liver regeneration. Hepatology. 2018;68(5):1977–90. 29704254 10.1002/hep.30067

[15] Fozza C, Longinotti M. T-cell receptor repertoire usage in hematologic malignancies. Crit Rev Oncol Hematol. 2013;86(3):201–11. 23219015 10.1016/j.critrevonc.2012.11.005

[16] Zelle-Rieser C, et al. T cells in multiple myeloma display features of exhaustion and senescence at the tumor site. J Hematol Oncol. 2016;9(1):116. 27809856 10.1186/s13045-016-0345-3 PMC5093947

[17] Lagreca I, et al. The role of T-cell immunity in monoclonal gammopathy and multiple myeloma: from immunopathogenesis to novel therapeutic approaches. Int J Mol Sci. 2022;23(9):5242. 35563634 10.3390/ijms23095242 PMC9104275

[18] Schrama D, Ritter C, Becker JC. T cell receptor repertoire usage in cancer as a surrogate marker for immune responses. Semin Immunopathol. 2017;39(3):255–68. 28074285 10.1007/s00281-016-0614-9

[19] Rubtsova K, et al. Many different vbeta CDR3s can reveal the inherent MHC reactivity of germline-encoded TCR V regions. Proc Natl Acad Sci U S A. 2009;106(19):7951–6. 19416894 10.1073/pnas.0902728106 PMC2674405

[20] Jia Q, et al. Diversity index of mucosal resident T lymphocyte repertoire predicts clinical prognosis in gastric cancer. Oncoimmunology. 2015;4(4):e1001230. 26137399 10.1080/2162402X.2014.1001230 PMC4485732

[21] Gerlinger M, et al. Ultra-deep T cell receptor sequencing reveals the complexity and intratumour heterogeneity of T cell clones in renal cell carcinomas. J Pathol. 2013;231(4):424–32. 24122851 10.1002/path.4284 PMC4241038

[22] Han Y, et al. Identification of characteristic TRB V usage in HBV-associated HCC by using differential expression profiling analysis. Oncoimmunology. 2015;4(8):e1021537. 26405574 10.1080/2162402X.2015.1021537 PMC4570125

[23] Cha E, et al. Improved survival with T cell clonotype stability after anti-CTLA-4 treatment in cancer patients. Sci Transl Med. 2014;6(238):238ra70. 24871131 10.1126/scitranslmed.3008211 PMC4558099

[24] Postow MA, et al. Peripheral T cell receptor diversity is associated with clinical outcomes following ipilimumab treatment in metastatic melanoma. J Immunother Cancer. 2015;3:23. 26085931 10.1186/s40425-015-0070-4 PMC4469400

[25] Rustad EH, et al. Stability and uniqueness of clonal immunoglobulin CDR3 sequences for MRD tracking in multiple myeloma. Am J Hematol. 2019;94(12):1364–73. 31571261 10.1002/ajh.25641 PMC7449571

[26] Puig N, et al. The predominant myeloma clone at diagnosis, CDR3 defined, is constantly detectable across all stages of disease evolution. Leukemia. 2015;29(6):1435–7. 25567133 10.1038/leu.2015.7

[27] Kim JA, et al. CDR3 spectratyping identifies clonal expansion within T-cell subpopulations that demonstrate therapeutic antitumor activity. Surgery. 2004;136(2):295–302. 15300194 10.1016/j.surg.2004.05.003

[28] Fozza C, et al. Study of the T-cell receptor repertoire by CDR3 spectratyping. J Immunol Methods. 2017;440:1–11. 27823906 10.1016/j.jim.2016.11.001

[29] Fozza C, et al. Patients with early-stage myelodysplastic syndromes show increased frequency of CD4 + CD25 + CD127(low) regulatory T cells. Acta Haematol. 2012;128(3):178–82. 22890368 10.1159/000339498

[30] Langerak AW, et al. Molecular and flow cytometric analysis of the Vbeta repertoire for clonality assessment in mature TCRalphabeta T-cell proliferations. Blood. 2001;98(1):165–73. 11418476 10.1182/blood.v98.1.165

[31] Lu J, et al. Analysis of T-cell repertoire in hepatitis-associated aplastic anemia. Blood. 2004;103(12):4588–93. 14988156 10.1182/blood-2003-11-3959

[32] Fozza C, et al. Patients with myelodysplastic syndromes display several T-cell expansions, which are mostly polyclonal in the CD4(+) subset and oligoclonal in the CD8(+) subset. Exp Hematol. 2009;37(8):947–55. 19409953 10.1016/j.exphem.2009.04.009

[33] Bazarbachi AH, et al. Relapsed refractory multiple myeloma: a comprehensive overview. Leukemia. 2019;33(10):2343–57. 31455853 10.1038/s41375-019-0561-2

[34] Gooding S, et al. Multiple cereblon genetic changes are associated with acquired resistance to lenalidomide or pomalidomide in multiple myeloma. Blood. 2021;137(2):232–7. 33443552 10.1182/blood.2020007081 PMC7893409

[35] Chanan-Khan AA, et al. Pomalidomide: the new immunomodulatory agent for the treatment of multiple myeloma. Blood Cancer J. 2013;3(9):e143. 24013664 10.1038/bcj.2013.38 PMC3789204

[36] Dingli D, et al. Therapy for relapsed multiple myeloma: Guidelines from the Mayo Stratification for Myeloma and Risk-adapted therapy. Mayo Clin Proc. 2017;92(4):578–98. 28291589 10.1016/j.mayocp.2017.01.003 PMC5554888

[37] Dimopoulos MA, et al. Multiple myeloma: EHA-ESMO clinical practice guidelines for diagnosis, treatment and follow-up(†). Ann Oncol. 2021;32(3):309–22. 33549387 10.1016/j.annonc.2020.11.014

[38] Moreau P, et al. Treatment of relapsed and refractory multiple myeloma: recommendations from the international myeloma working group. Lancet Oncol. 2021;22(3):e105-18. 33662288 10.1016/S1470-2045(20)30756-7

[39] Callander NS, et al. NCCN Guidelines^®^ insights: multiple myeloma, version 3.2022. J Natl Compr Canc Netw. 2022;20(1):8–19. 34991075 10.6004/jnccn.2022.0002

[40] Gandhi AK, et al. Immunomodulatory agents lenalidomide and pomalidomide co-stimulate T cells by inducing degradation of T cell repressors ikaros and aiolos via modulation of the E3 ubiquitin ligase complex CRL4CRBN. Br J Haematol. 2014;164(6):811–21. 24328678 10.1111/bjh.12708 PMC4232904

[41] Kravtsov DS, et al. Roles of CD4 + T cells as mediators of antitumor immunity. Front Immunol. 2022;13:972021. 36159781 10.3389/fimmu.2022.972021 PMC9500154

[42] Quach H, et al. Mechanism of action of immunomodulatory drugs (IMiDS) in multiple myeloma. Leukemia. 2010;24(1):22–32. 19907437 10.1038/leu.2009.236 PMC3922408

[43] Ríos-Tamayo R, et al. Pomalidomide in the treatment of multiple myeloma: design, development and place in therapy. Drug Des Devel Ther. 2017;11:2399–408. 28860711 10.2147/DDDT.S115456 PMC5574598

[44] Muthu Raja KR, et al. Increased T regulatory cells are associated with adverse clinical features and predict progression in multiple myeloma. PLoS ONE. 2012;7(10):e47077. 23071717 10.1371/journal.pone.0047077 PMC3468567

[45] Feng X, et al. Targeting CD38 suppresses induction and function of T regulatory cells to mitigate immunosuppression in multiple myeloma. Clin Cancer Res. 2017;23(15):4290–300. 28249894 10.1158/1078-0432.CCR-16-3192 PMC5540790

[46] Kalff A, et al. Planned withdrawal of dexamethasone after pomalidomide low-dose dexamethasone induction for lenalidomide-refractory multiple myeloma (ALLG MM14). Haematologica. 2022;107(1):321–5. 34587718 10.3324/haematol.2021.278655 PMC8719089

[47] Ravi G, Costa LJ. Bispecific T-cell engagers for treatment of multiple myeloma. Am J Hematol. 2023;98(Suppl 2):S13–21. 35702871 10.1002/ajh.26628

[48] D’Souza C, Prince HM, Neeson PJ. Understanding the role of T-cells in the antimyeloma effect of immunomodulatory drugs. Front Immunol. 2021;12:632399. 33746969 10.3389/fimmu.2021.632399 PMC7973099

[49] Gorski J, et al. Circulating T cell repertoire complexity in normal individuals and bone marrow recipients analyzed by CDR3 size spectratyping. correlation with immune status. J Immunol. 1994;152(10):5109–19. 8176227

[50] Ochsenreither S, et al. Relative quantification of TCR vbeta-chain families by real time PCR for identification of clonal T-cell populations. J Transl Med. 2008;6:34. 18593466 10.1186/1479-5876-6-34 PMC2467404

[51] Ciupe SM, et al. Quantification of total T-cell receptor diversity by flow cytometry and spectratyping. BMC Immunol. 2013;14:35. 23914737 10.1186/1471-2172-14-35 PMC3750526

[52] Muraro PA, et al. Rapid identification of local T cell expansion in inflammatory organ diseases by flow cytometric T cell receptor vbeta analysis. J Immunol Methods. 2000;246(1–2):131–43. 11121554 10.1016/s0022-1759(00)00309-4

[53] Vujović M, et al. Signatures of T cell immunity revealed using sequence similarity with TCRDivER algorithm. Commun Biol. 2023;6(1):357. 37002292 10.1038/s42003-023-04702-8 PMC10066310

