# Supplementary material for: Pomalidomide in patients with multiple myeloma: potential impact on the reconstitution of a functional T-cell immunity
Source: Immunol Res. 2024 Sep 24;72(6):1470–8. doi: 10.1007/s12026-024-09546-w (PMC11618177; doi:10.1007/s12026-024-09546-w)

**Supplementary Figure 1**

**Gating strategy for the analysis of V Beta in CD4 and CD8 lymphocytes**


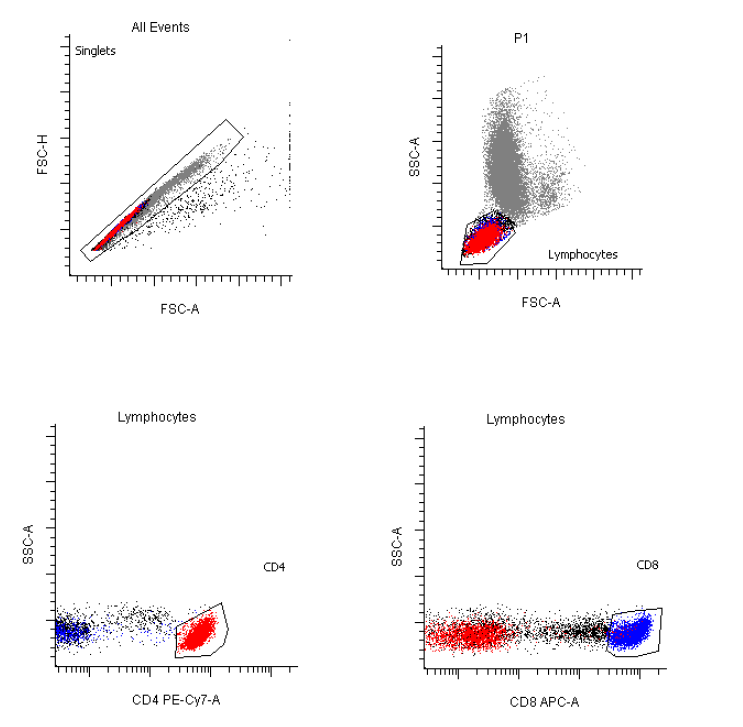


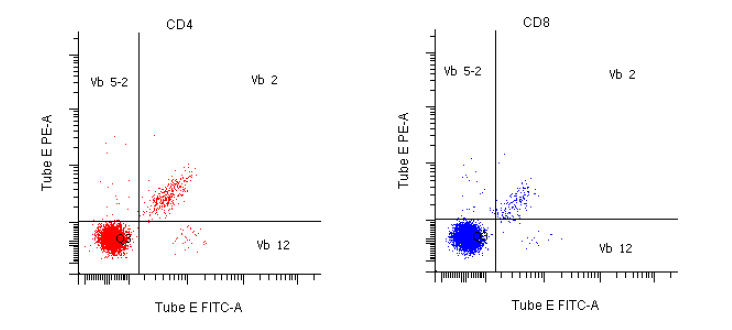


**Gating strategy for the analysis of B, T, Nk, T NK and TCR alpha-beta and TCR gamma-delta lymphocytes**


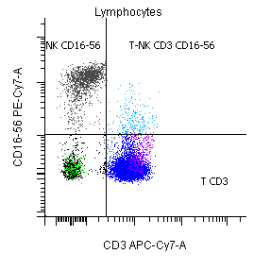

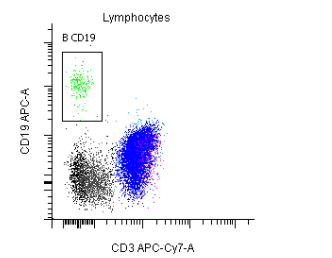


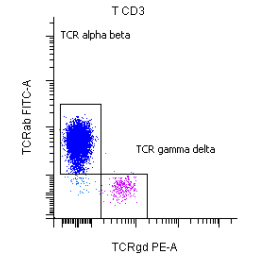


**Gating strategy for the analysis of T Reg lymphocytes**


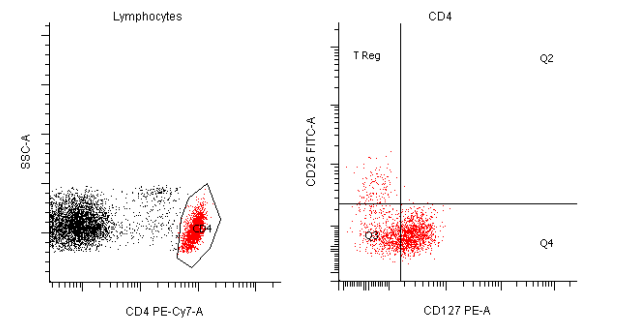

Supplement: Supplementary file 1 — (DOCX 221 KB) [file 12026_2024_9546_MOESM1_ESM.docx]
