# Supplementary material for: Pomalidomide in patients with multiple myeloma: potential impact on the reconstitution of a functional T-cell immunity
Source: Immunol Res. 2024 Sep 24;72(6):1470–8. doi: 10.1007/s12026-024-09546-w (PMC11618177; doi:10.1007/s12026-024-09546-w)
Supplement: Supplementary file 2 — (DOCX 22.0 KB) [file 12026_2024_9546_MOESM2_ESM.docx]

**Supplementary Table 1** Patients' characteristics (n=10), therapeutic lines, routine blood values and therapeutic response.

| Sex | Age  (years) | HB  (gr/dL) | WBC  (10/μl) | PLT  (K/μl) | ISS | Monoclonal Component | Previous Therapy | Time intervals (months) from 1st treatment to POMA | Treatment response | | |
| --- | --- | --- | --- | --- | --- | --- | --- | --- | --- | --- | --- |
|  |  |  |  |  |  |  |  |  | ***t=2*** | ***t=3*** | ***t=4*** |
| F | 67 | 13.3 | 3860 | 141,000 | III | Normal | VD-RD-BVD- | 46 | PD | NA | NA |
| M | 73 | 7.7 | 2600 | 130,000 | III | NA | VMP-RD-BVD- | NA | PD | NA | NA |
| F | 71 | 9.6 | 1740 | 164,000 | II | Normal | VELCADE-SCT-VEL-DEX-RD- | 29 | PD | NA | NA |
| F | 70 | 13.2 | 4340 | 149,000 | II | Normal | VMP-RD- | 23 | PR | PR | VGPR |
| F | 68 | 13.3 | 5120 | 197,000 | II | Normal | VMP-VD-RD- | 26 | MR | SD | SD |
| F | 61 | 11 | 4900 | 241,000 | II | Normal | VED-SCT-RD- | 107 | PR | PD | NA |
| M | 78 | 14 | 5500 | 155,000 | I | Normal | VD-VMP-RD- | 58 | PR | PR | VGPR |
| M | 70 | 13.8 | 5230 | 157,000 | I | Normal | VMP-RD- | 16 | PR | PR | VGPR |
| M | 78 | 11.9 | 5400 | 224,000 | III | Normal | VAD-VD-VMP-RD-BUD- | 210 | NA | SD | SD |
| M | 74 | 9 | 38000 | 48,000 | II | Normal | VTD-SCT-RD-KRd- | 68 | SD | VGPR | CR |

F:female: M: male: ISS: internationa staging system; Therapy: VD = bortezomib + dexamethaone; RD = Lenalidomide + plus low-dose dexamethasone; BVD = Bendamustine + bortezomib + low-dose dexamethasone; VMP = bortezomib + melphalan + prednisone; VELCADE = Velcade (Bortezomib); SCT = stem cell transplantation; VEL-DEX = Velcade (Bortezomib) + dexamethaone; VED = Vincristine + epirubicin + dexamethasone; VAD = Vincristine + doxorubicin + dexamethasone; BUD = Oral budesonide; VTD = Bortezomib + thalidomide + dexamethasone; KRd = Carfilzomib + lenalidomide + low-dose dexamethasone; HB: Haemoglobin; WBC: white blood cell; PLT: platelets. T: time. Different treatment response: Progressive Disease (PD), Partial Response (PR), Complete Response (CR), Very Good Partial Response (VGPR), Minimal Response (MR), and Stable Disease (SD).
