# Supplementary material for: Pomalidomide in patients with multiple myeloma: potential impact on the reconstitution of a functional T-cell immunity
Source: Immunol Res. 2024 Sep 24;72(6):1470–8. doi: 10.1007/s12026-024-09546-w (PMC11618177; doi:10.1007/s12026-024-09546-w)
Supplement: Supplementary file 3 — (DOCX 18.4 KB) [file 12026_2024_9546_MOESM3_ESM.docx]

**Supplementary Table 2** All antibodies used in the study.

| **Antibody** | **Clone** | **Fluorochrome** | **Manifacture** |
| --- | --- | --- | --- |
| Anti-TCR alpha/beta | WT31 | FITC | BD Biosciences |
| Anti-TCR gamma/delta | 11F2 | PE | BD Biosciences |
| CD3 | SK7 | APC-Cy 7 | BD Biosciences |
| CD4 | SK3 | PerCP-Cy 5.5 | BD Biosciences |
| CD4 | SK3 | PE-Cy 7 | BD Biosciences |
| CD8 | SK1 | APC | BD Biosciences |
| CD16 | B73.1 | PE-Cy 7 | BD Biosciences |
| CD19 | SJ25C1 | APC | BD Biosciences |
| CD25 | M-A251 | FITC | BD Pharmigen |
| CD45 | 2D1 | PerCP-Cy 5.5 | BD Biosciences |
| CD45 | 2D1 | APC-Cy 7 | BD Biosciences |
| CD56 | NCAM16.2 | PE-Cy 7 | BD Biosciences |
| CD127 | HIL-7R-M21 | PE | BD Pharmigen |
|  |  |  |  |
| **IOTest® Beta Mark PN IM3497 TCR Vβ Repertoire Kit** |  |  | Beckman Coulter |
| **Tube A** |  |  |  |
| Vb 5.3 (TRBV5-5) | PE 3D11 | PE | Beckman Coulter |
| Vb 7.1 (TRBV4-1, TRBV4-2, TRBV4-3) | ZOE | PE+FITC | Beckman Coulter |
| Vb 3 (TRBV28) | CH92 | FITC | Beckman Coulter |
| **Tube B** |  |  |  |
| Vb 9 (TRBV3-1) | FIN9 | PE | Beckman Coulter |
| Vb 17 (TRBV19) | E17.5F3 | PE+FITC | Beckman Coulter |
| Vb 16 (TRBV14) | TAMAYA1.2 | FITC | Beckman Coulter |
| **Tube C** |  |  |  |
| Vb 18 (TRBV18) | BA62.6 | PE | Beckman Coulter |
| Vb 5.1 (TRBV5-1) | IMMU157 | PE+FITC | Beckman Coulter |
| Vb 20 (TRBV30) | ELL1.4 | FITC | Beckman Coulter |
| **Tube D** |  |  |  |
| Vb 13.1 (TRBV6-5, TRBV6-6, TRBV6-9) | IMMU222 | PE | Beckman Coulter |
| Vb 13.6 (TRBV6-6) | JU74.3 | PE+FITC | Beckman Coulter |
| Vb 8 (TRBV12-3, TRBV12-4) | 56C5.2 | FITC | Beckman Coulter |
| **Tube E** |  |  |  |
| Vb 5.2 (TRBV5-6) | 36213 | PE | Beckman Coulter |
| Vb 2 (TRBV20-1) | MPB2D5 | PE+FITC | Beckman Coulter |
| Vb 12 (TRBV10-3) | VER2.32 | FITC | Beckman Coulter |
| **Tube F** |  |  |  |
| Vb 23 (TRBV13) | AF23 | PE | Beckman Coulter |
| Vb 1 (TRBV9) | BL37.2 | PE+FITC | Beckman Coulter |
| Vb 21.3 (TRBV11-2) | IG125 | FITC | Beckman Coulter |
| **Tube G** |  |  |  |
| Vb 11 (TRBV25-1) | C21 | PE | Beckman Coulter |
| Vb 22 (TRBV2) | IMMU546 | PE+FITC | Beckman Coulter |
| Vb 14 (TRBV27) | CAS1.1.3 | FITC | Beckman Coulter |
| **Tube H** |  |  |  |
| Vb 13.2 (TRBV6-2) | H132 | PE | Beckman Coulter |
| Vb 4 (TRBV29-1) | WJF24 | PE+FITC | Beckman Coulter |
| Vb 7.2 (TRBV4-3) | ZIZOU4 | FITC | Beckman Coulter |
